# Nek family of kinases in cell cycle, checkpoint control and cancer

**DOI:** 10.1186/1747-1028-6-18

**Published:** 2011-10-31

**Authors:** Larissa Moniz, Previn Dutt, Nasir Haider, Vuk Stambolic

**Affiliations:** 1 Department of Medical Biophysics, University of Toronto, Toronto, Ontario, M5G 2M9, Canada; 2 Ontario Cancer Institute/University Health Network, Toronto, Ontario, M5G 2M9, Canada; 3 Centre for Cell Signalling, Barts Cancer Institute, Queen Mary University of London (QMUL), London EC1M 6BQ, UK

**Keywords:** Nek family, cell cycle, checkpoint control, cilia, cancer

## Abstract

Early studies in lower Eukaryotes have defined a role for the members of the NimA related kinase (Nek) family of protein kinases in cell cycle control. Expansion of the Nek family throughout evolution has been accompanied by their broader involvement in checkpoint regulation and cilia biology. Moreover, mutations of Nek family members have been identified as drivers behind the development of ciliopathies and cancer. Recent advances in studying the physiological roles of Nek family members utilizing mouse genetics and RNAi-mediated knockdown are revealing intricate associations of Nek family members with fundamental biological processes. Here, we aim to provide a comprehensive account of our understanding of Nek kinase biology and their involvement in cell cycle, checkpoint control and cancer.

## Introduction

Deregulation of the cell cycle is a hallmark of neoplastic transformation and plays a central role in the initiation and progression of cancer. The fidelity of the cell cycle is tightly maintained by numerous regulatory proteins, most notably kinases. Cyclin dependent kinases (CDK), in complex with their partner cyclins, are considered the master regulators of the cell cycle. Members of the Aurora and Polo families are also critical components of the cell cycle machinery. More recently, the NimA related kinase (Nek) family protein kinases begun to emerge as important players in regulation of the eukaryotic cell cycle both during normal cell cycle progression and in response to genotoxic stress. This review aims to provide a systematic account of our understanding of Nek kinase biology and their involvement in disease drawn from biochemical, cell biology, animal model and genetic studies.

### Nek kinase family

The filamentous fungus *Aspergillus nidulans *Never in mitosis A (NimA) is the founding member of the (NEK) family of serine-threonine kinases, and an essential regulator of mitosis [1,2]. NimA is required for transport of active CDC2 into the nucleus thus allowing initiation of mitosis [3]. Moreover, NimA promotes mitotic chromosome condensation through phosphorylation of histone H3 at serine 10 and may regulate nuclear membrane fission during mitotic exit [4,5].

The critical role for NimA in promoting cell cycle progression in *A.nidulans *raised the possibility that homologues of NimA existed in higher eukaryotes. Consistent with this, overexpression of NimA in *S.pombe *and in human HeLa cells induced chromosome condensation in the absence of other mitotic events, such as the microtubule spindle assembly or Cdc2 activation [6,7]. Indeed, NimA-related kinases have been identified throughout higher eukaryotes with a significant expansion of the family through evolution. While a single NimA homologue exists in yeast, 2, 4 and 11 NimA-related kinases were identified in *D.melanogaster, C.elegans *and mammals respectively.

**Figure 1:**
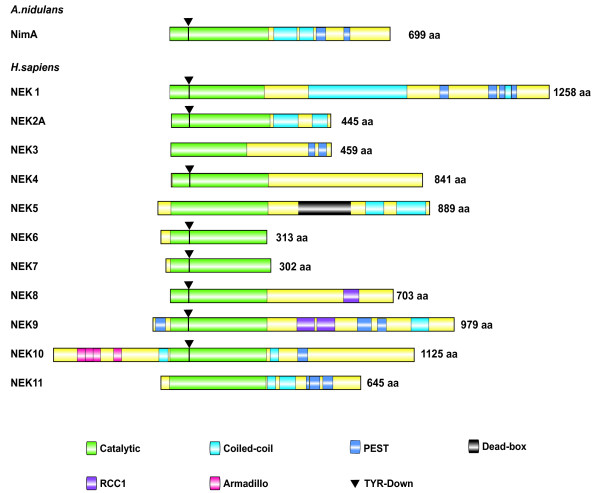
**Alignment of the key structural features of the 11 mammalian NIMA-related kinases and the fungal *Aspergillus *NIMA kinase**. The relative positions of significant motifs and regions are indicated.

NimA consists of an N-terminal catalytic domain, coiled-coiled domains, which mediate oligomerization, and PEST sequences, which participate in ubiquitin-dependent proteolysis, a process that may be required for *A.nidulans *to exit mitosis [8] (Figure 1). NimA kinase activity exhibits a preference for N-terminal hydrophobic residues and a phenylalanine at position -3 relative to the phosphorylated residue (FR/KR/KS/T, target residue underlined) [9]. Despite low overall sequence homology, the organizational features of NimA are broadly conserved among mammalian Nek kinases. For instance, all Nek kinases except Nek10 contain N-terminal catalytic domains, whereas Nek4, 6 and 7 are the only family members that do not contain coiled-coiled motifs. Moreover 6 of 11 mammalian Nek kinases contain putative PEST sequences (Figure 1).

Outside regions of homology, certain Nek kinases contain unique protein domains that point to the acquistion of novel functions relative to the ancestral NimA protein. Nek8 and Nek9 contain regulator of chromosome condensation (RCC1) repeats, which are homologous to RCC1, a guanine nucleotide exchange factor (GEF) for the small GTPase, Ras-related nuclear protein (Ran). While the role of the RCC1 domain has not been characterized in Nek8, in Nek9 this domain acts as a negative regulator of Nek9 catalytic activity and can interact with Ran. However, there is no evidence that Nek9 can act as a GEF towards Ran [10]. Additional unique domains in Nek family members include a predicted DEAD-box helicase-like domain in Nek5 and a cluster of armadillo repeats in Nek10 (Figure 1).

A recent determination of the three dimensional structure of Nek7 revealed a novel autoinhibitory sequence within the kinase domain. This tyrosine-down motif within the nucleotide binding lobe projects into the active site of the kinase, generating an inactive conformation. Activation of Nek6/7 occurs in two distinct ways, by interaction with Nek9's non-catalytic C-terminal tail, which relieves the autoinhibition, and by direct Nek9-mediated phosphorylation within the activation loop [10,11]. An equivalent autoinhibitory tyrosine can be found in 8 of 11 Nek kinases (including Nek2 and Nek6) (Figure 1), and 10% of all human kinases [11].

A divergence in function between mammalian Neks and the ancestral NimA is highlighted by the fact that only nim-1 from the related fungus *Neosporra crassa *can functionally complement the nimA mutation [12]. Neither the yeast nimA homologues (fin1 in *S.pombe*; KIN3 in *S.cervisae*) nor Nek2, the closest mammalian nimA homologue, are able to rescue the cell cycle defect incurred by defects in nimA [13,14]. While mammalian Nek kinases do not phenocopy the NimA mutation, they are involved in many aspects of cell cycle progression. Notably, many of these functions can be attributed to the regulation of microtubules and microtubule containing structures. More recently, several Nek family members have also been shown to participate in control of cell cycle checkpoints following cellular stress and DNA damage, as well as development of cancer.

### Nek kinases, microtubules and microtuble-based organelles

#### a) Nek2 in control of centrosome splitting

Based on sequence homology within the kinase domain, Nek2 is the closest mammalian NimA homologue. Unlike NimA however, Nek2 is not essential for mitotic entry, but instead regulates centrosome separation during mitosis [15,16]. Nek2 localizes to centrosomes during interphase and early mitosis where it interacts with and phosphorylates several centrosomal proteins including cNap-1, Rootletin and β-catenin [16-19]. Nek2 localization and ability to phosphorylate c-Nap and Rootletin is mediated by interaction with members of the Hippo pathway, Mst2 and hSav1 [20]. Inhibition of Nek2 catalytic activity or knockdown of its' substrates, cNap-1, Rootletin or β-catenin, inhibits centrosome separation, spindle assembly and formation of multinucleated cells [15,18-20]. In addition to the centrosome, Nek2 localizes to the condensed chromatin, the midbody and the kinetochores of dividing NIH3T3 cells [21]. Significantly, knockdown of Nek2 causes displacement of the centromeric protein Mad2 from the kinetochores and impairs chromosome segregation [21]. Taken together, these studies indicate that Nek2 may coordinate cell division on multiple levels.

A fundamental role of Nek2 in control of the cell cycle progression and division is strongly corroborated by its function in early embryogenesis. Downregulation of Nek2 in one-cell mouse embryos through microinjection of dsRNA prevented 75% of the embryos from reaching the blastocyst stage, with most arresting at the four-cell stage [22,23]. Most embryos displayed morphological defects in both mitotic and interphase blastomeres, forming abnormal spindle structures and displaying irregular nuclear morphologies, including dumbbell shaped nuclei, nuclear bridges, and micronuclei.

#### b) Nek 6, 7 and 9 and the mitotic spindle

Nek6 and Nek7 are highly related and are almost entirely composed of catalytic domains, which share 87% identity. While they were originally identified based on their ability to phosphorylate p70 S6 kinase in vitro, the physiological significance of this interaction remains unclear [24,25]. Instead, Nek6 and Nek7 were found to act downstream of Nek9 and regulate the mitotic spindle and cytokinesis [26]. Specifically, Nek6 or Nek7 depletion led to fragile spindle formation during mitosis and prolonged the activation of the spindle assembly checkpoint (SAC) preventing progression to anaphase [26]. In addition to regulation of spindle formation, Nek6/7 contribute to the final stage of cell division, as cells that are treated with pharmacological inhibitors of the SAC continue to progress through mitosis but arrest again during cytokinesis [26]. Consistent with these findings, Nek9 function in spindle dynamics has also been demonstrated, whereby inhibition of Nek9 through microinjection of α-Nek9 antibodies impaired spindle assembly and chromosome alignment during metaphase [10]. Finally, Nek6, 7 and 9 have recently been implicated in centrosome splitting [27]. In HeLa cells, Nek9 is activated by sequential phosphorylation by CDK1 and PLK1 during mitosis, which leads to Nek6/7-dependent phosphorylation of Eg5, and its accumulation at centrosomes, an event required for centrosome separation [27].

Taken together, these cell-based studies suggested that Nek6/7/9 might be critical for regulation of microtubule organization during mitosis. Indeed, targeted disruption of the Nek7 gene in mice revealed that this kinase was indispensable for murine development, with only rare homozygous-null animals surviving to one month of age [28]. At birth, Nek7-deficient mice weighed slightly less than their littermates, but thereafter exhibited severe growth retardation, weighing roughly half as much as their littermates by twenty days of age. Furthermore, Nek7^-/- ^MEFs were frequently found to be bi/multinuclear or mononuclear with enlarged nuclei. Analysis of metaphase chromosome spreads revealed increased polyploidy and genetic instability leading to aneuploidy. Evidence of multi-centrosomes in the binucleated cells, as well as more frequent incidence of chromosomal lagging and bridges at anaphase or telophase were further indicative of cytokinesis failures. Interestingly, judged by the strong phenotypes elicited by Nek7 deletion, despite their strong homology, Nek6 could not compensate for loss of Nek7 in both cultured cells and the whole organism. This may in part be explained by differential tissue distribution and subcellular localization of Nek6 and 7 [26,29].

In addition to Nek6, 7 and 9, Nek3 and Nek4 are also implicated in control of microtubule dynamics. For example, in post-mitotic neurons, expression of a Nek3 mutant lacking the regulatory phosphorylation site (T475) within the PEST sequence, believed to act as a dominant negative, resulted in disruption of microtubule deacetylation, polarity and overall neuronal morphology [30]. Finally, knockdown of Nek4 in MCF7 cells altered the cellular sensitivity to the microtubule poisons taxol and vincristine, suggesting that Nek4 may also regulate microtubule dynamics [31].

#### c) Nek1, Nek8 and Ciliagenesis

Nek kinases prominently feature in the biology of cilia, which are microtubule-based organelles that are structurally and functionally similar to flagella (reviewed in [32]). Two types of cilia exist. The motile cilia function to move extracellular fluid and debris and are found on certain cell types such as the tracheal epithelia where they work to sweep debris out of the airway. On the other hand, primary cilia are present on most cell types and coordinate the cellular responses with the extracellular environment. Primary cilia form during interphase from the mother centriole and dissemble prior to mitosis (reviewed in [33,34]). Ciliary protein mutations are the basis of a number of human genetic disorders termed ciliopathies, including retinal degeneration, polycystic kidney, liver and pancreatic diseases, abnormalities in neural tube closure and skeletal defects (reviewed in [35]).

Nek kinases were first linked with ciliagenesis with the discovery that mutations in Nek1 and Nek8 are the causal events in independent mouse models of polycystic kidney disease (PKD) [36,37]. The Kat and Kat2J strains, harbor mutations in the NEK1 gene that result in production of truncated Nek1 proteins. Mice carrying these mutations display facial dysmorphism, dwarfing, male sterility due to testicular hypoplasia and reduced spermatogenesis, anemia, and progressive polycystic kidney disease [38,39]. Another model of PKD is the Jck mouse strain, which harbors a G448V missense mutation in the C-terminal RCC1 domain of NEK8 [36,40,41]. The Kat, Kat2J and the Jck strains recapitulate the characteristics of PKD seen in humans to varying degrees, with the phenotype of the Jck mice, in particular, strongly resembling the autosomal dominant human disease. Specifically, Jck mice recapitulate many of the hallmark features of the human condition, including onset and sites of the disease, as well as the abnormal epidermal growth factor receptor (EGFR) expression and increased cAMP signaling [41]. Recently, loss-of-function Nek1 mutations in 2 families were identified and found to be the underlying cause of the ciliopathy, autosomal-recessive short-rib polydactyly syndrome [42].

*In vitro *work with cultured cells has provided further insight into the roles of Nek1 and Nek8 in ciliagenesis. In wildtype kidney epithelial cells, Nek8 localizes to primary cilia, while in cells derived from Jck mice, mutant NEK8 exhibits cytoplasmic and perinuclear localization, which correlates with increased cilia length [41]. In Jck mice, the expression of the polycystins PC-1 and PC-2 is elevated and while they are ordinarily restricted to the basal bodies of wild-type cilia, both proteins are found along the length of the cilia of kidney cells [43]. Notably, the accumulation of polycystins in cilia has been reported in other polycystic kidney disease models and mutations in PC-1 and PC-2 themselves can lead to PKD [44]. In the case of Nek1, a role in cilia formation was demonstrated in IMCD3 cells. Overexpression of Nek1 in these normally ciliated cells derived from the inner medullary collecting duct of the murine kidney, led to inhibition of ciliagenesis [45]. This is likely dependent on Nek1 catalytic activity, as a catalytically inactive mutant of Nek1 while localizing to cilia failed to affect cilia formation [45].

It has been proposed that the ability to coordinate the primary cilium with the cell cycle coevolved with the expansion of the Nek family [34]. For example, *A.nidulans *and yeast are non-ciliated and only contain a single NimA-related kinase. In *D.melanogaster *and *C.elegans*, which have 2 and 4 NimA-related kinases respectively, ciliated cells are terminally differentiated and thus do not coordinate cilia function with the cell cycle. In contrast, organisms such as mammals, *Chlamydomonas *and *Tetrahymena *which feature proliferating ciliated cells display an expansion of the Nek family, as they contain 11, 10 and 35 members respectively [34].

### Nek Kinases and Checkpoint Control

In addition to the established functions during mitosis, certain Nek kinases also participate in cell cycle regulation following genotoxic stress. All eukaryote cells have multiple molecular mechanisms to identify and repair damaged DNA and preserve genomic integrity (reviewed in [46]). An important aspect of this process is activation of a checkpoint and induction of cell cycle arrest, to allow the cell time to repair damage. Cell cycle arrest can be triggered at G1/S, intra-S and G2/M phases of the cell cycle following damage caused by endogenous sources, such as stalled replication forks, or by exogenous agents, including ultraviolet (UV) radiation, ionizing radiation (IR), reactive oxygen species (ROS) and certain chemotherapeutic agents. Upon successful repair, the cell will re-enter the cell cycle.

Checkpoint activation is initiated by the PIKK family serine/threonine kinases ATM (ataxia-telangiectasia mutated) and ATR (ATM and rad3-related), and their effector kinases Chk1/2 (checkpoint kinase 1/2). Parallel to Chk1/2 signaling, p38 MAPK and it's downstream kinase MK2 (MAPK activated protein kinase 2) have also been identified as key regulators of cell cycle arrest (reviewed in [47]). Ultimately, the two checkpoint pathways culminate in inactivation of CDKs.

Some of the key molecular targets that mediate checkpoint engagement are the transcription factor p53 and the CDK-activating phosphatases Cdc25A, B and C. Activation of the ATM/ATR/Chk1/2 cascade leads to stabilization of p53, and subsequent upregulation of a number of antiproliferative genes, including p21 [48-53]. While p53 likely contributes to all checkpoints it is absolutely required for the G1/S cell cycle arrest. Many human tumors and immortalized cell lines exhibit compromised p53 activity and G1/S arrest following damage. In such cells, the G2/M checkpoint takes on increasing importance for maintaining genomic stability. Cdc25A, B and C are inactivated via phosphorylation by mutiple kinases, including Chk1/2 and Nek11 (reviewed in [54,55]). Following genotoxic stress, Cdc25A undergoes ubiquitin-mediated degradation, which occurs in a Chk1/2-dependent manner [56,57]. On the other hand, Chk1/2 and/or MK2 phosphorylation of Cdc25B and C leads to association with 14-3-3 and their cytoplasmic sequestration, away from their targets CDKs [58-61].

Amongst the Nek family, Nek11 contribution to checkpoint control has been best characterized. Meliexetian et al. demonstrated that in response to IR, Nek11 gets activated via phosphorylation on S273 by the ATM effector kinase, Chk1, which also phosphorylates Cdc25A on S76, priming it for further phosphorylation within the DSG motif [55]. Significantly, Nek11 acted as the Cdc25A DSG motif kinase promoting its ubiquitination and degradation. Consistent with this, HeLa cells depleted for Nek11 display elevated levels of Cdc25A protein and fail to undergo IR-induced G2/M arrest [55].

Nek1 and Nek2 also participate in IR-induced checkpoints. For instance, IR of Cos-7 cells results in reduction of Nek2 catalytic activity, likely in an ATM/protein-phosphatase-1 (PP-1)-dependent manner, integral to the IR-induced inhibition of centrosome splitting [62]. Unlike Nek2, in HK2 and HeLa cells, Nek1 expression and catalytic activity are elevated in response to IR [63]. Highlighting the importance of Nek1 levels following IR, Nek1^-/- ^cells displayed defective G1/S and G2/M checkpoints and were unable to repair their DNA, leading to accumulation of double strand breaks [64]. Nek1 subcellular localization is also regulated by IR. While in unstimulated cells Nek1 is predominantly cytoplasmic, following treatment with various genotoxic agents including IR, UV, etoposide and cisplatin, Nek1 localizes to γ-H2AX positive nuclear foci [63,64]. Significantly, unlike Nek11 and Nek2, IR-induced changes in Nek1 activity and localization occur independently of ATM/ATR [65].

Work from our laboratory on Nek10, a previously uncharacterized Nek family member, has uncovered its role in G2/M checkpoint control [66]. In response to UV irradiation, HEK293 and MCF10A cells depleted for Nek10 displayed an impaired G2/M arrest. Intriguingly, these studies revealed that Nek10 can promote autoactivation of MEK1 in response to UV irradiation, but not mitogenic stimuli. While ectopic expression of Nek10 enhanced, its depletion inhibited UV-induced MEK1/2 and ERK1/2 phosphorylation. Nek10 was shown to interact with both Raf-1 and with MEK1 in a Raf-1-dependent manner. Surprisingly, Raf-1 was required for Nek10 complex formation with MEK1, but its catalytic activity was dispensable for activation of MEK1 in response to UV irradiation. Instead, MEK1 underwent auto-activation upon exposure to UV irradiation. Integrin-stimulated MEK1 autophosphorylation has previously been described in the context of cell adhesion [67], but unlike the response to UV irradiation, it required prior phosphorylation at S298 by PAK1.

Regardless of the nature of the upstream signal, MEK1 autoactivation represents an alternate means of ERK pathway activation. Significantly, ERK1/2 activation has been linked to checkpoint control upon genotoxic stress, as well as recovery from cell cycle arrest and DNA repair [68-71]. MEK1 autophosphorylation can be detected following UV irradiation, as well as other stressors such as anisomycin and sorbitol treatment, but not following EGF or PMA stimulation (Moniz L. and Stambolic V., unpublished observation) consistent with the notion that MEK autoactivation occurs in stimulus-specific manner. Other means of communication between Nek kinases and the ERK signalling cascade may also exist. For instance, during the first meiotic prophase, Nek2 activity is sensitive to U0126/MEK inhibition, while in vitro it can be phosphorylated and activated by p90Rsk2, a downstream target of ERK1/2 [72]. Moreover, Nek2A directly interacts with ERK2 and may specify its localization to centrosomes [73].

### Involvement of Nek Family Members in Cancer

Sequencing and resequencing of cancer genomes has identified mutations of several Nek family members. Cancer-associated mutations in Nek kinase genes appearing in the COSMIC database, a curated catalogue of somatic mutations identified in sequenced tumors or cancer cell lines maintained by the Sanger Institute/Wellcome Trust, are listed in Table 1. As additional data from sequencing projects is incorporated, further mutations will undoubtedly be uncovered. Nevertheless, it remains to be determined which, if any, of the observed mutations represent driver mutations that actively promote oncogenesis, and which are merely passenger mutations created by the genetic instability endemic to tumorigenesis. In addition to the mutation data, a series of studies are documenting direct involvement of Nek family members in cell transformation and tumorigenesis. The following section summarizes results of such studies.

**Table 1:** Functions of mammalian Nek kinases

ID	Functions	Disease associations	Cancer Mutations
**Nek1**	-excess catalytic activity leads to loss of primary cilia (IMCD3 cells) [45] -Nek1^-/- ^cells display defective G1/S and G2/M checkpoints and DNA repair after IR [64]	-mutation is causal in Kat, Kat2J mouse models of PKD [37] -mutation identified as causal in 2 families with autosomal-recessive short-rib polydactyly syndrome [42]	*tumor samples:* ovarian (C191F, K779N) large intestinal (N181N*) lung (E25K) *cultured cells:* lung (A294P)

**Nek2**	-promotes centrosome splitting at G2/M and chromosome segregation [15,16,73,15,16,21] -regulates chromosome segregation [73] -catalytic activity inhibited following IR, preventing centrosome separation [62]	-elevated expression in: colangiocarcinoma tumors [74,75] - elevated expression in MDA-MB-231 and MCF7 cells; knockdown suppresses proliferation *in vitro *and tumor burden of xenografts *in vivo *[75]	*tumor samples:* breast (R115Q, E278K) stomach (G134D)

**Nek3**	-regulates microtubule deacetylation in neurons [30] -regulates prolactin-mediated cytoskeleton rearrangement and motility of T47D breast cancer cells [84]		*tumor samples:* ovarian (D413Y) *cultured cells:* stomach(Y398**)

**Nek4**	- knockdown alters sensitivity of MCF7 cells to microtubule poisons taxol and vincristine [31]		*tumor samples:* large intestinal (R777K)

**Nek5**	-uncharacterized		*tumor samples:* mouth (K201**)

**Nek6**	-required for mitotic spindle formation and cytokinesis [26] -promotes centrosome separation [27] -activated downstream of Nek9 [85]	-overexpressed in a variety of human tumors [77] -knockdown inhibits HeLa xenografts [77] -Nek6 expression downregulated following p53-induced senescence [78]	*tumor samples:* ovarian (Y295C, Y291Y*) *cultured cells:* kidney (I99S)

**Nek7**	-required for mitotic spindle formation and cytokinesis [26] -promotes centrosome separation [27] -activated downstream of Nek9 [85]	-rare Nek7^-/- ^mice survive to birth and exhibit severe growth retardation [28]	*tumor samples:* lung (G7**) ovarian (I275M) *cultured cells:* stomach (M8L, V285I)

**Nek8**	-regulation of primary cilia; regulates localization and expression of ciliary proteins PC-1, PC-2 [40,43]	-mutation is causal in Jck mouse model of PKD [36] -overexpressed in primary human breast tumors [86]	*tumor samples:* liver (G605D) ovarian (P703***) stomach (R292Q) *cultured cells:* pancreatic (A197P) skin (L621F) stomach (L341P)

**Nek9**	-regulation of primary cilia; regulates localization and expression of ciliary proteins PC-1, PC-2 [40,43]		*tumor samples:* ovarian (V319***) *cultured cells:* lung (P870S) stomach (V631I, R786Q)

**Nek10**	- regulates establishment of UV-induced G2/M checkpoint [66] - interacts with Raf-1/MEK1 to promote MEK1 autoactivation following UV irradiation [66]	-GWAS identified Nek10 as a strong breast cancer susceptibility locus [80] - various mutations identified in primary tumours and cancer cells lines [82]	*tumor samples:* ovarian (V319***) *cultured cells:* lung (P870S) stomach (V631I, R786Q)

**Nek11**	-required for G2/M arrest following IR [55] -activated by Chk1 phosphorylation -phosphorylates DSG motif of Cdc25A leading to its ubiquitination and degradation [55]		*tumor samples:* ovarian (A66V, V568I, D875Y, F50L, S852S*), lung (R878M) brain (I783V, S797S*) *cultured cells:* skin (E379K)

Nek kinase somatic mutations in tumor samples and cultured cells as catalogued in the COSMIC database (Sanger Institute/Wellcome Trust). (*) silent mutations; (**) nonsense mutations; (***) deletion-frameshift mutations.

#### Nek1

Renal tubular epithelial cells established from Kat2J mice exhibit abnormal nuclear morphologies including multinuclei, micronuclei, and bridging chromosomes [65]. Multipolar spindles, lagging chromosomes, improper chromosome movements, and incomplete cytokinesis were also observed during mitosis. As a consequence of these mitotic defects, populations of Kat2J cells manifest progressively worsening aneuploidy, with three quarters of cells having greater than 4N DNA content after several passages. Indicative of their transformation, xenograft injection of Kat2J mutant, but not wild-type renal tubular cells led to formation of tumors [65]. Consistently, 89% of mice heterozygous at the Kat2J locus (Nek1^+/-^) developed lymphomas between 17 and 24 months of age, compared to 30% of wild-type mice [65]. Importantly, lymphoma cells were devoid of Nek1 immunoreactivity, suggestive of loss of heterozygosity at this locus.

#### Nek2

Elevated levels of Nek2 has been found in certain human cancers, raising the possibility that they may represent potential therapeutic targets. Colangiocarcinoma is an aggressive cancer originating in the liver bile duct epithelium with a markedly poor clinical prognosis. A cDNA array analysis comparing gene expression in colangiocarcinoma and normal liver tissue revealed Nek2 upregulation in these tumors, which was further confirmed in a subsequent evaluation of seven colangiocarcinoma cell lines [74]. Significantly, siRNA-mediated knockdown of Nek2 in xenografts generated by femoral injection of HuCCT1 colangiocarcinoma cells, attenuated cancer progression. Similar observations were made in several breast cancer cell lines, both ER-positive and ER-negative [75]. Namely siRNA-mediated knockdown of Nek2 in MCF7, MDA-MB-231 and Hs578T mammary carcinoma cell lines suppressed their proliferation, invasiveness, and anchorage-independent growth *in vitro *[75]. Further, Nek2 siRNAs significantly reduced tumor burden in mice femorally injected with either MCF7 (ER-positive) or MDA-MB-231 (ER-negative) cells [75].

Elevated Nek2 expression has also been noted in colorectal cell lines, as well as in tumor biopsies [76]. Similar to the effects in breast cancer cell lines, Nek2 siRNA impaired the in vitro proliferation of the DLD-1 and Colo320 carcinoma cell lines, as well as xenografts generated by injection of DLD-1 cells [76]. Finally, Nek2 siRNA and Cisplatin displayed an additive suppressive effect in treating DLD-1 xenografts, suggesting a possible therapeutic opportunity in targeting Nek kinases [76].

#### Nek6

Similar to Nek2, Nek6 is overexpressed in tumors from a variety of tissues including breast, uterus, colon, ovary, thyroid, and cervix, as well as a number of associated carcinoma cell lines [77]. A recent study linking Nek6 to p53-induced senescence has shed light on how Nek6 may promote tumorigenesis. In both human lung fibroblasts and EJ human bladder carcinoma cells, Nek6 expression decreased upon p53-induced senescence [78]. Importantly, ectopic expression of Nek6 in EJ cells reduced markers of senescence, including cell-cycle arrest, production of reactive oxygen species (ROS) and senescence-associated β-galactosidase activity caused by expression of p53 expression or treatment with chemotherapeutic agents such as doxorubicin [78,79]. Consistently, knockdown of Nek6 suppressed anchorage-independent growth of several carcinoma cell lines, including colon (HCT-15), stomach (NCI-N87) and cevix (HeLa), as well as growth of HeLa xenografts [77].

#### Nek10

A potential association of Nek10 and cancer was uncovered by a comprehensive genome wide association study (GWAS) involving over 37,000 breast cancer samples and 40,000 controls, which identified a strong breast cancer susceptibility locus within human chromosome 3p24 (p value = 4.1 × 10-23) [80]. Importantly, the sub-region of 3p24 identified by this GWAS contains only two genes, Nek10 and the solute carrier family 4, sodium bicarbonate co-transporter, member 7 (SLC4A7) [80]. Interestingly, this susceptibility locus associates with increased risk of breast cancer for BRCA2 but not BRCA1 mutation carriers [81].

Nek10 may also be subject of direct mutations in cancer. Namely, whole genome sequencing of 210 primary tumors and immortalized human cancer cell lines uncovered more than a 1000 somatic mutations within the coding sequences of the 518 predicted human protein kinases [82,83]. One parameter for distinguishing driver and passenger mutations is the ratio of non-synonymous to synonymous mutations appearing in distinct cancers. In this regard Nek10 is noteworthy in having thirteen catalogued missense mutations in six cancers. Based on mutation frequency, Nek10 was defined as one of 120 kinases predicted to contain a driver mutation [82]. This raises the possibility that disrupted Nek10 function contributes to oncogenesis, though this remains to be formally tested through rigorous experimentation. Of note, Nek10 mutations were found with the same frequency (4/33) as the mutations of B-Raf and liver kinase B1 (LKB1), kinases previously firmly implicated in tumorigenesis [82]. Nek10 mutations were found in both primary tumors (ovarian (A66K, V568I, D875Y, F50L), lung (R878M), brain (I783V)) and cultured cell lines (skin (E379K), lung (P1115L), pancreas (D665Y, stomach (R878K, R103C)) [82]. While none of the identified mutations map to the catalytic domain of Nek10, their effect on protein function is currently unknown.

### Summary

Early phenotypic analyses of the mutant fungi for the archetypal Nek kinase revealed their involvement in cell cycle regulation. Subsequent studies in yeast and frogs, and more recently mice, have uncovered the fascinating intricacy in the control of the cell cycle and its checkpoints by various members of the Nek family. Further, mutations of Nek family members have also been identified as drivers behind the development of ciliopathies and cancer. Recent emergence of comprehensive cancer genomes is highlighting certain members of the Nek family as targets of frequent mutations. Despite remarkable progress in understanding the biology of the Nek family, the most interesting work is yet to be done, fuelled by the advent of gene knockout, RNAi-mediated knockdown, naturally occurring mutant and xenograft tumor models.

## Competing interests

The authors declare that they have no competing interests.

## Authors' contributions

LM, PD, NH and VS wrote the manuscript, read and approved of the final manuscript.
